# Prevalence and Associated Factors of Chronic Depression Among Older Adults: A Systematic Review, Meta‐Analysis, and Meta‐Regression

**DOI:** 10.1002/gps.70160

**Published:** 2025-10-18

**Authors:** M. W. Stratmann, H.‐H. König, A. Hajek

**Affiliations:** ^1^ Department of Health Economics and Health Services Research University Medical Center Hamburg‐Eppendorf Hamburg Germany

**Keywords:** chronic depression, dysthymia, meta‐analysis, old age, persistent depressive disorder, prevalence, systematic review

## Abstract

**Objectives:**

Although late‐life depression is common, there has previously not been a comprehensive review of chronic depression (CD) in older adults. This systematic review summarizes prevalence rates, potential risk factors, and consequences of CD in later life.

**Methods:**

This preregistered review (PROSPERO: CRD42025649324) searched MEDLINE, Web of Science, CINAHL and PsycINFO from inception to February 2025. Observational studies reporting the prevalence of CD in older adults (mean age 60+) were included. Study quality was assessed using the Joanna Briggs Institutes Critical Appraisal Tool. Random‐effects models were used to estimate pooled prevalence across subgroups and meta‐regression analyses were used to explore sources of heterogeneity.

**Results:**

A total of 39 articles (38 studies) met the inclusion criteria; 20 articles (27 data points) were included in meta‐analysis for point prevalence. Overall point prevalence was 4.02% [2.88%–5.35%], with estimates varying by assessment method: 2.30% [1.47%–3.31%] using DSM‐IV/DSM‐III‐R, 7.12% [2.31%–14.22%] using DSM‐III/ICD‐10% and 5.52% [3.80%–7.54%] using rating scales. Prevalence varied also by region and was higher for women. Consistent risk factors included less physical activity and higher impairment in daily life. Evidence on consequences was sparse.

**Conclusions:**

Approximately 4% of adults aged 60+ meet criteria for CD. Estimates vary substantially by method and region, and potential risk factors and outcomes remain poorly understood. Future studies should target underrepresented subgroups ‐ such as the oldest old, the institutionalized and physically and cognitively impaired individuals—using both dimensional and categorical assessment. CD in late life appears often unrecognized, untreated and underresearched.

## Key Points

- Around 4% of older adults meet criteria for chronic depression
- Prevalence estimates vary widely between studies
- Classification system and region of study explain some heterogeneity
- Consistent risk factors are lower physical activity and greater impairment

## 1 Introduction

Depression in older adults, also called late‐life depression, is frequent [1, 2, 3] and has a complex relationship with physical health [4], leading to negative outcomes such as frailty [5], accelerated aging [6], and overall morbidity [3, 7]. Approximately one third of depressive disorders are chronic [8, 9, 10], and rates of chronicity might even be higher in older populations, reaching up to 50% [11]. Chronic depression (CD) is associated with substantial individual and societal burden [9, 10, 12, 13].

In the Diagnostic and Statistical Manual of Mental Disorders, Fifth Edition (DSM‐V) [14], CD is defined as “Persistent Depressive Disorder” (PDD) combining mild, severe and fluctuating forms of depression over a period of at least 2 years and subsuming chronic forms previously coded separately [15].

While research has often focused on CD in younger adults, older adults face distinct risk factors, such as bereavement, chronic illness, loneliness, and geriatric giants (e.g., frailty, cognitive decline) [16, 17, 18]. Although this may increase vulnerability to CD, older adults might also better tolerate stressors due to developed coping strategies [18], leading to uncertainty about whether CD is more or less prevalent in this age group.

Furthermore, depression might present itself differently in late‐life [16, 19], often marked by somatic symptoms, cognitive changes and loss of interest [20]. These age‐specific symptoms along with complex depression trajectories may result in diagnostic challenges and treatment gaps [16]. Although estimates of episodic late‐life depression range widely (7.2% [2] to 35.1% [1] depending on the usage of clinical diagnoses or screening tools), studies suggest that once depression occurs in old age, it often persists due to low remission rates [21, 22].

Despite its clinical relevance, CD in older adults has received limited attention. A prior meta‐analysis on mental disorders in late‐life [23] included only one study, reporting a life‐time prevalence of dysthymia of just 1.3% [24]. To date, there has been no comprehensive meta‐analysis of prevalence of CD in older adults. Further, risk factors specific to late‐life may differ (e.g., greater importance of insomnia) [20, 25] and aggregated evidence is sparse.

There has been no systematic review/meta‐analysis on this topic previously. Thus, the aim of this research is to meta‐analytically summarize existing studies on the prevalence of CD in older adults and to describe potential risk factors and consequences. Thereby it aims to provide a more accurate prevalence estimate than individual studies, uncover correlates regarding risk for CD and consequences, and identify research gaps to inspire further research. The meta‐regression can provide deeper insights into which factors might influence the prevalence of CD.

## 2 Materials and Methods

This study adheres to the Preferred Reporting Items for Systematic Reviews and Meta‐Analysis (PRISMA) guidelines [26] and was preregistered (PROSPERO: CRD42025649324).

### 2.1 Search

On 07/02/2025 four electronic databases were searched: MEDLINE, Web of Science, CINAHL and PsycINFO. The search strategy was developed in close collaboration with a university librarian and focused on identifying articles that included terms related to: (1) CD, (2) older adults and (3) prevalence information. The complete search strategy (for all databases) can be found in the Supporting Information S1: (Tables 1–4). Furthermore, articles were identified via extensive manual backward and forward citation search.

### 2.2 Screening

Two reviewers (MWS & ML) independently screened (a) all titles/abstracts of all identified articles and (b) all full‐texts of retrieved articles. A pre‐test involving 100 titles/abstracts was conducted. Inclusion criteria remained unchanged. At both screening stages, disagreements were resolved through discussion or a third party (AH) if necessary.

### 2.3 Eligibility Criteria

The inclusion criteria were defined as follows:

‒ Population‐representative observational studies: cross‐sectional or longitudinal (at least two measurements over the course of at least 2 years)
‒ Individuals aged at least 60 years or older (mean age) or reporting of results for the respective age bracket
‒ Reporting of prevalence of CD according to accepted clinical criteria (see 2.4.).
‒ Utilization of appropriate tools for assessing CD
‒ Language: English, German or Polish
‒ Published in peer‐reviewed scientific journals

Exclusion criteria were:

‒ Studies focusing on any disease‐specific samples: for example, CD in older adults with dementia, cancer or frailty
‒ Samples taken solely in special environments (e.g., nursing homes)
‒ Gray literature

There were no restrictions regarding to year of publication and publication location.

### 2.4 Definition of Chronic Depression

We define CD in line with the DSM‐V definition of PDD combining mild, severe and fluctuating forms of depression over a period of at least 2 years and subsuming chronic forms (e.g., dysthymia, double depression) previously coded separately [14].

For comparison in subgroup meta‐analysis, we grouped classifications systems:

‒ DSM‐V [14]: PDD including each subtype: pure dysthymia, persistent major depressive episode, intermittent major depressive episodes with current episode, and intermittent major depressive episode without current episode.
‒ DSM‐III‐R [27], DSM‐IV [15] and DSM‐IV‐TR [28]: dysthymia, chronic major depression, double depression, recurrent depressive disorder with incomplete remission between episodes.
‒ DSM‐III [29] and ICD‐10 [30]: dysthymia/dysthymic disorder
‒ Depressive rating scales (e.g., Center for Epidemiologic Studies Depression Scale (CES‐D; [31]): prospectively assessing clinically significant depressive symptoms over the course of at least 2 years)

### 2.5 Data Extraction

Data was extracted by one author (MWS) and very carefully verified by a second (ML). In cases of missing data, we contacted authors via e‐mail. Key information (country, data sample, study design, time of data collection, classification system, assessment tool, condition, age, proportion of women and sample size) and main outcomes (type of prevalence (e.g., point, 12‐month, life‐time), prevalence of CD (in percent and as absolute number), and associated factors) were extracted. In cases where multiple studies reported on the same dataset, we opted for the study that employed the larger sample size or gave more comprehensive results (e.g., stratified by gender or age).

### 2.6 Quality Appraisal

The Joanna Briggs Institutes (JBI) standardized critical appraisal instrument for prevalence studies [32] was used by two reviewers (MWS & ML) independently to evaluate the study quality. A third party (AH) was involved if necessary. The instrument assigns scores from 0 to 9, with higher values indicating better study quality and a lower risk of bias. There was no cut‐off score for the exclusion of studies for the meta‐analysis.

### 2.7 Statistical Analysis

The meta‐analysis focused on point prevalence, with separate analyses for 12‐month and life‐time prevalence reported in the Supporting Information S1. The random‐effects model was chosen because we expect the prevalence to vary substantially between studies (e.g., time of data collection and location).

In this meta‐analysis, estimates from all classification systems are pooled. To control and compare case definitions, meta‐analysis was conducted by subgroups for classification systems applied: DSM‐V, DSM‐IV/DSM‐III‐R (including DSM‐IV‐TR), DSM‐III/ICD‐10, and rating scales. We argue that pooling is justified in order to approximate the comprehensive DSM‐V definition of PDD. Studies only reporting on pure dysthymia (excluding chronic major depression, double depression and recurrent depression without full remission) may underestimate CD [13]. While estimates based on screening tools (longitudinal studies) may somewhat overestimate prevalence, it has been argued that these measures are less prone to recall bias and misattribution of depressive symptoms to physical illness (as in standardized interviews) [33, 34]. Consequently, we believe that categorical and dimensional estimates provide complementary estimates.

Additional meta‐analyses were conducted separately for both men and women to investigate gender differences. Analysis by age group was planned but not enough data was available. Heterogeneity between studies was evaluated using the *I* ^*2*^ statistic, with values ranging of 25%–50% classified as “low”, 50%–75% as “moderate”, and 75% or more as “high” heterogeneity [35].

Further, we conducted a random‐effects meta‐regression to explore the sources of heterogeneity. Classification system, risk of bias (quality appraisal score) and region were included. Mean age and proportion of women were not included due to the amount of missing data.

We assume that the publication of prevalence rates—particularly from large epidemiological studies—is less prone to publication bias, as these studies often report all collected outcomes, including non‐significant results, and are typically conducted with pre‐specified protocols [36]. Still, a funnel plot and Egger test (*p* < 0.05 signals the presence of publication bias) were conducted and are reported in detail in the Supporting Information S1.

All analyses were conducted using Stata 18.5 [37]. The data and the syntax have been made available (https://osf.io/ncrx9/).

### 2.8 Potentially Associated Factors

Relevant effect sizes were extracted and are reported in detail in the Supporting Information S1. While meta‐analysis on associated factors would have been desirable, necessary conditions were not met: namely, comparable effect sizes, consistent definitions and operationalizations, similar adjustments, and, above all, a sufficient number of studies reporting on the same factors. Data aggregation of evidence thus remains descriptive, and risk factors are reported as “consistently associated” if several studies have found associations in the same direction, “probably associated” if a few studies have found associations in the same direction, “not associated” if no studies found associations, and “inconclusive” if results are mixed or the number of studies is insufficient. The results thus roughly describe the state of the evidence without making statements about the magnitude of associations.

## 3 Results

### 3.1 Search Results

**FIGURE 1:**
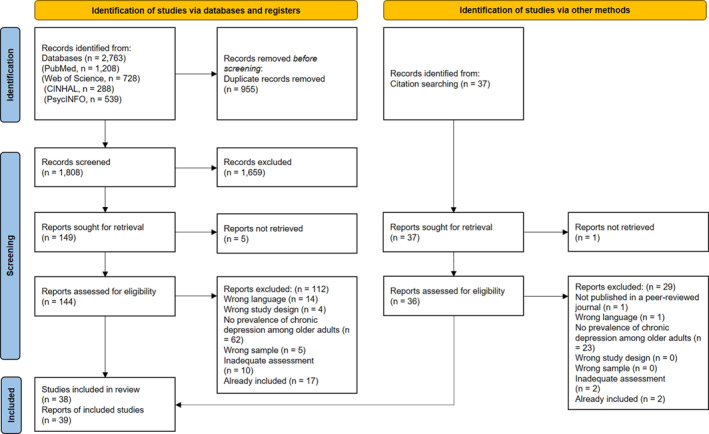
PRISMA flow chart.

The search yielded a total of 2763 articles through data bases (Figure 1). After removing duplicates, 1808 titles/abstracts were screened. Of these, 144 full‐texts were assessed for eligibility, with most exclusions due to missing reporting of prevalence data on CD in older adults. Additionally, 37 articles were identified through a manual citation search. Of these, 36 articles were retrieved and 29 excluded.

In total, 39 articles (covering 38 studies) were included [19, 33, 38, 39, 40, 41, 42, 43, 44, 45, 46, 47, 48, 49, 50, 51, 52, 53, 54, 55, 56, 57, 58, 59, 60, 61, 62, 63, 64, 65, 66, 67, 68, 69, 70, 71, 72, 73, 74].

### 3.2 Study Overview

Key study information are shown in Table 1. Data was from Europe (*n* = 14), Asia (*n* = 11), North America (*n* = 9), South America (*n* = 2), Africa (*n* = 1) and Oceania (*n* = 1). Cross‐sectional design was utilized in 28 studies and longitudinal design in 10 studies.

**TABLE 1:** Description and results of included studies.

Author (Year)	Country	Sample; design	Time of data collection	Diagnostic system; assessment tool	Condition	Mean age (SD) or [age bracket]	Female %	Sample size (*n*)	Prevalence % (*n*)
Andreas et al. (2017) [39]	Europe	MentDis_ICF65+ study; cross‐sectional	NR	DSM‐IV; CIDI	D	73.7 (5.6)	50.7	3142	LT: 2.9 (91) 12M: 2.9 (91) P: 2.7 (85)
	Italy							518	LT: 3.1 (16) 12M: 3.1 (16) P: 2.9 (15)
	Spain							555	LT: 2.5 (14) 12M: 2.5 (14) P: 2.6 (14)
	England							496	LT: 3.4 (17) 12M: 3.4 (17) P: 2.9 (14)
	Germany							511	LT: 2.5 (13) 12M: 2.5 (13) P: 2.5 (13)
	Israel							542	LT: 5.2 (28) 12M: 5.2 (28) P: 4.3 (23)
	Switzerland							520	LT: 4.0 (21) 12M: 4.0 (21) P: 3.8 (20)
	Netherlands	Longitudinal aging Study Amsterdam (LASA)							
Beekman et al. (2001) [40]		Longitudinal	1992–1996	Rating; CES‐D ≥ 16 (20‐items)	PDS over 4 years	[55–85]	53.0	2200	P: 6.5 (143)
Beekman et al., (2004) [41]		Cross‐sectional	1992–1993	DSM‐IV; DIS	D	[55–85]	57.9	646	LT: 4.61 (30)
Bendayan et al. (2020) [42]	USA	Health and retirement sample (HRS); longitudinal	1998–2006 (w4–w8)	Rating; CES‐D ≥ 4 (8‐items)	PDS (at least 3 waves)	69.4 (3.2)	59.7	3466	P: 2.1 (73)
Bendayan et al. (2020) [42]	Europe	Survey of health, aging and retirement in Europe (SHARE); longitudinal	2004–2013 (w1–w5, expect w3)	Rating; EURO‐D ≥ 4	PDS (at least 3 waves)			3940	P: 7.3 (286)
	European mediterranean (Spain, France, Italy, and Israel)					69.5 (3.1)	55.6	1587	P: 11.0 (174)
	Non‐mediterranean (Sweden, Denmark, Netherlands, Germany, Belgium, Switzerland, and Austria)					69.3 (3.1)	53.1	2353	P: 4.8 (112)
Blanco et al. (2010) [43]	USA	National epidemiologic survey on alcohol and related conditions (NESARC) 2001; cross‐sectional	2001–2002	DSM‐IV; AUDADIS‐IV	Chronic major depressive disorder and D	[65+]	NR	6999	LT: 4.0 (278)
Bland et al. (1988) [44]	Canada	NR; cross‐sectional	1983–1986	DSM‐III; DIS	D	[55+]	NR	721	LT: 5.1 (37)
Byers et al. (2010) [45]	USA	National comorbidity survey ‐ replication (NCS‐R); cross‐sectional	2001–2003	DSM‐IV; CIDI	D	68.0 (9.2)	59.0	2575	12M: 0.8 (21)
Carta et al. (1995) [46]	Italy	Health in sardinia; cross‐sectional	NR	DSM‐III‐R; CIDI	D	[65+]	NR	NR	LT: 6.4 (NR)
Chong et al. (2012) [47]	Singapore	Singapore Mental health study (SMHS); cross‐sectional	2009–2010	DSM‐IV; CIDI	D	[65+]	NR	412	LT: 0.0 (0)
Costa et al. (2007) [33]	Brazil	Bambuí health aging study (BHAS); cross‐sectional	2001	ICD‐10; SCAN	D and double depression	79.9 (NR)	62.5	392	P: 9.0 (33)
Fichter et al. (1995) [48]	Germany	Munich Study on the oldest old; cross‐sectional	1990	DSM‐III‐R; GMS	D	[85+]	77.1	353	12M: 5.1 (18)
Forsell et al. (1998) [49]	Sweden	NR; cross‐sectional	1992	DSM‐IV; CPRS	D	84.6 (4.5)	77.6	1101	P: 3.5 (39)
Heok et al. (1996) [50]	Singapore	NR; cross‐sectional	1992	DSM‐III‐R; GMS, AGECAT	D	[65+]	57.0	1062	P: 0.2 (2)
Huang et al. (2019) [51]	China	China mental health survey (CHMS); cross‐sectional	2013–2015	DSM‐IV; SCID, CIDI	D	[65+]	54.6	5326	12M: 1.7 (91)
Ihara et al. (1998) [52]	Japan	NR; cross‐sectional	1993	DSM‐III‐R; SCID	D	72.8	58.1	1965	P: 0.5 (10)
Isometsä et al. (1997) [53]	Finland	Use of health services and health status; cross‐sectional	1994	DSM‐III‐R; CIDI	D	[60–79]	53.2	556	P: 0.9 (5)
Keqing et al. (2008) [54]	China	NR; cross‐sectional	2004–2005	DSM‐IV; SCID	D	[60–69] [70+]	NR	NR	[60–69] P: 3.59 (NR) [70+] P: 3.52 (NR)
Kivelä et al. (1989) [55]	Finland	Ähtäri study 1984; cross‐sectional	1984–1986	DSM‐III; semi‐structured interviews based on HDRS and DSM‐III	D	[60+] Men: 69.7 (6.9) Women: 70.5 (7.4)	59.1	1235	P: 20.6 (199)
Kohn et al. (2008) [56]	Chile	Chile psychiatric prevalence study (CPSS); cross‐sectional	1992–1999	DSM‐III‐R; CIDI	D	[65+]	58.5	352	LT: 6.3 (22) 12M: 1.5 (5)
Kramer et al. (1985) [57]	USA	Eastern Baltimore Mental health survey (EBMHS); cross‐sectional	1980	DSM‐III; DIS	D	[65+]	62.0	923	6M: 1.0 (9)
Livne et al. (2018) [58]	USA	National epidemiologic survey on alcohol and related conditions (NESARC) 2012; cross‐sectional	2012–2013	DSM‐V; AUDADIS	PDD	[Birth cohort 1940–1946; around 67 years of age at data collection]	NR	3823	12M: 5.27 (201)
Lobo et al. (1995) [59]	Spain	Zaragoza city study; cross‐sectional	NA	DSM‐III‐R; GMS, AGECAT	D	74.9 (6.3)	59.7	1080	P: 1.3 (14)
Madianos et al. (1992) [60]	Greece	NR; cross‐sectional	NA	DSM‐III; PEF	D	74.0 (6.6)	63.2	251	P: 5.5 (14)
Markkula et al. (2015) [61]	Finland	Finnish health 2011 survey; cross‐sectional	2011	DSM‐IV; CIDI	D	[65+]	52.9	2237	12M: 5.0 (112)
Park et al. (2015) [62]	South‐Korea	Yeoncheon elderly depression and dementia study (YEDD); longitudinal	2008–2013	Rating; GDS ≥ 8 (short‐form; 15‐items)	PDS over 5 years	71.2 (5.0)	59.9	340	P: 8.8 (30)
Peltzer and Pengpid (2022) [63]	South‐Africa	International network for the demographic evaluation of populations and their health (INDEPTH); longitudinal	2014–2019	Rating; CES‐D ≥ 3 (8‐items)	PDS over 5 years	[60+]	0.00	2346	P: 3.3 (77)
Pengpid et al. (2023) [64]	Thailand	Health, aging, and retirement in Thailand (HART); longitudinal	2015–2017	Rating; CES‐D ≥ 10 (10‐items)	PDS over 2 years	[45–75+]	52.2	3390	P: 2.2 (76)
Penninx et al. (1998) [65]	USA	Established populations for epidemiologic studies of the elderly (EPESE); longitudinal	1982–1988	Rating; CES‐D ≥ 20(10; 11 and 20‐items)	PDS over 6 years	79.0 (NR)	64.6	4825	P: 3.0 (146)
Phillips et al., (2009) [66]	China	NR; cross‐sectional	2001–2005	DSM‐IV‐TR; SCID	D	[55+]	NR	16,253	P: 3.91 (635)
Regier et al. (1988) [67]	USA	Epidemiologic catchment area survey (ECA); cross‐sectional	1980–1984	DSM‐III; DIS	D	[65+]	NR	5702	P: 1.8 (103)
Reynolds et al. (2015) [68]	USA	National epidemiologic survey on alcohol and related conditions (NESARC) 2004; cross‐sectional	2004–2005	DSM‐IV; AUDADIS‐IV	D	[55–85+]	55.0	12,312	12M: 0.94 (138)
Schoevers et al. (2003) [69]	Netherlands	Amsterdam Study of the elderly (AMSTEL); longitudinal	NR	Rating; GMS, AGECAT	PDS over 3 years	[65–84]	NR	2244	P: 5.4 (122)
Skoog et al. (1993) [70]	Sweden	Gothenburg city study; cross‐sectional	NR	DSM‐III‐R; psychiatric evaluation	D	85.5 (NR)	71.5	494	P: 4.5 (22)
Subramaniam et al. (2020) [71]	Singapore	Singapore Mental health study 2 (SMHS‐2); cross‐sectional	2016	DSM‐IV; CIDI	D	[65+]	NR	1297	LT: 0.2 (3) 12M: 0.2 (3)
Teng et al. (2013) [72]	Taiwan	Survey of health and living status of the elderly in taiwan; longitudinal	1999–2007 (w1–w3)	Rating; CES‐D ≥ 10 (10‐items)	PDS over 8 years	[65+]	45.12	1784	P: 8.5 (152)
Wells et al. (2006) [73]	New Zealand	New Zealand mental health survey (NZMHS); cross‐sectional	2003–2004	DSM‐IV; CIDI	D	[65+]	NR	NR	12M: 0.4 (NR)
Zheng et al. (2018) [74]	England	English longitudinal study of aging (ELSA); longitudinal	2002–2004 (w1–w2)	Rating; CES‐D ≥ 4 (8‐items)	PDS over 2 years	65.2 (10.1)	57.0	7610	P: 6.9 (525)

*Note:* In some cases prevalence information was interpolated due to missing information. This applies, for example, to the calculation of the numerator if only the prevalence rate and the denominator were known. This approach may result in slight deviations between prevalence rates reported in the studies and those depicted here and utilized in the meta‐analysis.

Abbreviations: AGECAT, automated geriatric examination for computer assisted taxonomy; AUDADIS, alcohol use disorder and associated disabilities interview schedule; CES‐D, center for epidemiologic studies depression scale; CIDI, composite international diagnostic interview; CPRS, comprehensive psychopathological rating scale; D, dysthymia; DIS, diagnostic interview schedule; DSM, diagnostic and statistical manual of mental disorders; EURO‐D, euro depression scale; GDS, geriatric depression scale‐short form; GMS, geriatric mental state interview; HDRS, hamilton depression rating scale; ICD, international statistical classification of disease and related health problems, LT: life‐time prevalence; NR, not reported; SCAN, schedules for clinical assessment in neuropsychiatry; SCID, structured clinical interview for DSM; SD, standard deviation; P, point/1‐month prevalence; PDS, persistent depressive symptoms; PEF, psychiatric evaluation form; PDD, persistent depressive disorder; W, wave; 6M, 6‐month prevalence; 12M, 12‐month prevalence.

Publication date ranged from 1985 to 2023; only 4 studies were published in or after 2020. All data predated the COVID‐19 pandemic. Studies were based on representative surveys or large epidemiological studies with sample size varying from 251 [60] to 16,253 [66]. Mean age reported ranged from 65.2 [74] up to 85.5 years [70]. Most samples included 50%–65% women; two had more men, including one only‐men sample [63]. Three studies of the oldest old had over 70% women [48, 49, 70]. Cross‐sectional studies utilized the DSM‐III (*n* = 14), DSM‐IV (*n* = 13) and DSM‐III‐R (*n* = 7) and only one study utilized the DSM‐V [58]. Longitudinal studies primarily utilized some version of the CES‐D (*n* = 8) and operationalized CD as clinically significant depressive symptoms over a timeframe of 2 to up to 8 years. Most studies reported point prevalence; fewer reported 12‐month prevalence and life‐time prevalence, and one study reported 6‐month prevalence [57].

### 3.3 Quality Assessment/Risk of Bias

Quality assessment scores ranged from 5 to 9 (*M* = 7.26, SD = 0.94), indicating generally high quality and low risk of bias (Supporting Information S1: Table 5). All studies appropriately recruited participants and assessed depression, although definitions of CD varied. Common sources of bias included unexamined subgroup response rates (e.g., oldest old, individuals with disability), missing sample details and low response rates. Response rates varied from 20.0% in Germany [38] to 98.8% in Japan [52].

### 3.4 Meta‐Analysis and Meta‐Regression

**FIGURE 2:**
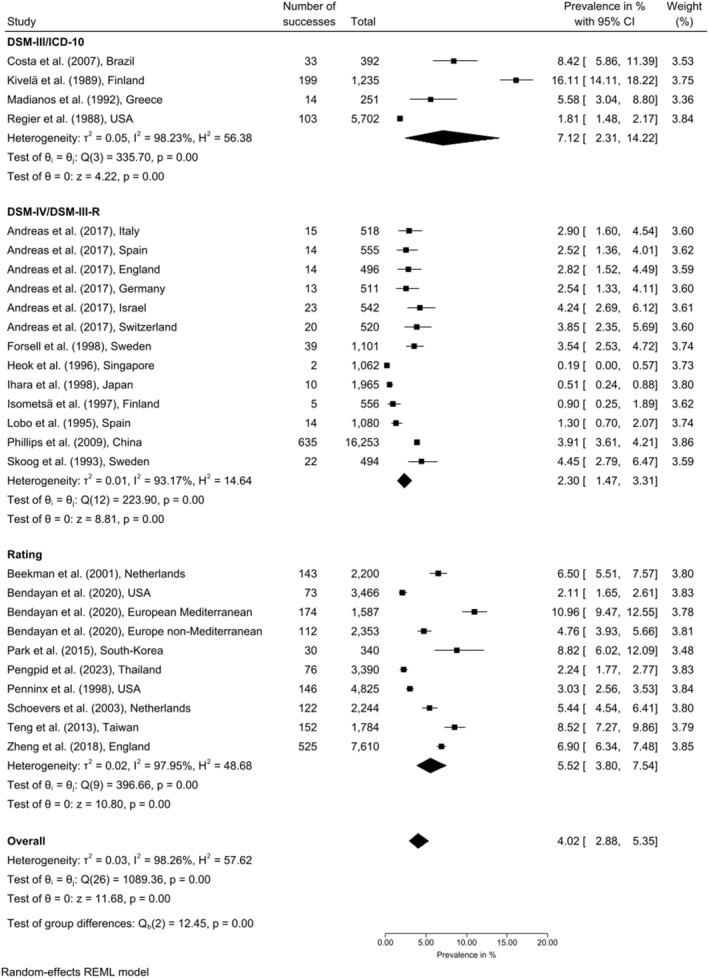
Random‐effects meta‐analysis for point prevalence of chronic depression in older adults by classification system.

Data from 20 articles including 27 data points were used in the meta‐analysis for point prevalence [33, 39, 40, 42, 49, 50, 52, 53, 55, 59, 60, 62, 64, 65, 66, 67, 69, 70, 72, 74]. Figure 2 shows estimates by classification system. Overall heterogeneity was high (*I* ^2^ > 90%). Pooled prevalence for adults aged 60 years or older was 4.02% [2.88%–5.35%]. Estimates varied: DSM‐III/ICD‐10 yielded 7.12% [2.31%–14.22%]; DSM‐IV/DSM‐III‐R 2.30% [1.47%–3.31%]; and rating scales 5.52% [3.80%–7.54%].

**FIGURE 3:**
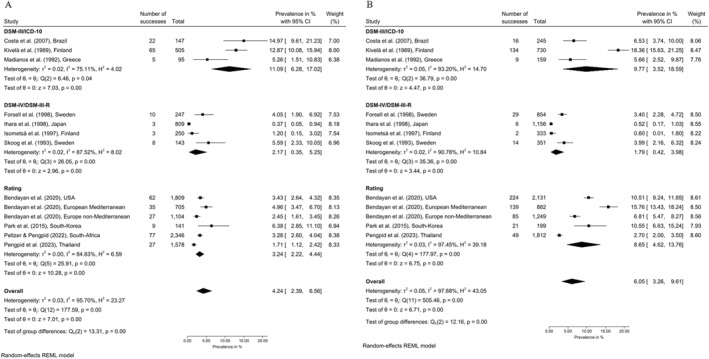
Random‐effects meta‐analysis for point prevalence of chronic depression in older men (A) and older women (B) by classification system.

Gender‐stratified estimates (Figure 3) showed 4.24% [2.39%–6.59%] for men and 6.05% [3.26%–9.61%] for women. Results differed by classification system for both men (DSM‐III/ICD‐10: 11.09% [6.28%–17.02%]; DSM‐IV/DSM‐III‐R: 2.17% [0.35%–5.25%]; rating scales: 3.24% [2.22%–4.44%]) and women (DMS‐III/ICD‐10: 9.77% [3.53%–18.59%]; DSM‐IV/DSM‐III‐R: 1.79% [0.42%–3.98%]; rating scales: 8.65% [4.62%–13.76%]).

Twelve‐month prevalence was 2.62% [1.71%–3.73%] and life‐time prevalence 2.97% [1.76%–4.48%] (Supporting Information S1: Figures 1 and 2).

Meta‐regression (Table 2) showed significantly higher prevalence with rating scales and DSM‐III/ICD‐10 compared to those based on DSM‐IV/DSM‐III‐R. Risk of bias scores were not associated. Prevalence rates found in North America were significantly lower compared to those in Europe; other continents did not differ significantly.

**TABLE 2:** Meta‐regression analysis of factors affecting heterogeneity in point prevalence of chronic depression.

Variables	Coefficient [95% confidence interval]	*p* value
**Diagnostic system** (DSM‐IV/DSM‐III‐R as reference)		
DSM‐III/ICD‐10	0.297 [0.137–0.458]	< 0.001
Rating	0.198 [0.093–0.303]	< 0.001
Risk of bias (From “0: Highest risk of bias” to “9: Lowest risk of bias”)	−0.469 [−0.111–0.017]	0.151
**Region** (Europe as reference)		
Asia	−0.064 [−0.170–0.042]	0.235
North America	−0.267 [−0.419−(−0.116)]	0.001
Other	−0.061 [−0.346–0.224]	0.675

*Note:* Number of observations = 27.

Funnel plot (Supporting Information S1: Figure 3) and Egger test (*p* = 0.68) indicated no publication bias (Supporting Information S1: Figure 3).

### 3.5 Risk Factors and Consequences

Eighteen studies investigated factors associated with CD. In the Supporting Information S1 complete information on extracted information is given (Supporting Information S1: Table 7), as well as a comprehensive summary (Supporting Information S1: Table 8).

Consistently associated risk factors included lower physical activity [62, 72, 74] and higher impairment in daily life [38, 65, 69, 72]. Probable risk factors included chronic physical illness [33, 40, 41, 64, 74] and comorbid major depressive disorder/double depression [33, 41] or anxiety [38, 69] and trauma history ([38, 41]). Factors that were consistently not associated were socioeconomic status, marital status, and cancer. Evidence for most other factors was inconclusive. Age‐related findings were mixed: one study found higher prevalence among the oldest old [38], others in younger old [65, 74], and many found no association [52, 62, 63, 64, 69, 72]. Gender effects were inconsistent across studies. For education, five studies found individuals with lower education background at greater risk [38, 41, 63, 72, 74], two those with higher education [55, 69] and three no connection [40, 62, 64]. Inconclusive evidence was further found for health behavior (e.g., obesity, alcohol, and smoking), psychosocial factors (e.g., social support, personal and family history of depression) and comorbidities including dementia. Some studies found CD to be associated with greater impairment due to cognitive decline [42, 49, 72, 74], while others did not [41, 48, 62].

Only a few studies reported on the consequences of CD. Andreas et al. [38] reported that more than half of dysthymic individuals never received any depression treatment. Kivelä et al. [55] found only 25% of dysthymic individuals were diagnosed with dysthymia prior to study participation. Teng et al. [72] reported a 66% increase in 4‐year mortality risk for individuals with CD after adjusting for known confounders. Analyses showed that mortality risk due to cardiovascular disease (e.g., ischemic heart disease, stroke) was raised for men and mortality risk due to non‐cardiovascular disease (e.g., cancer, diabetes) was raised for women.

## 4 Discussion

The study aimed to summarize the prevalence, risk factors, and consequences of CD in older adults. Overall, an estimated 4.0% of adults aged 60+ are affected, though prevalence varied depending on diagnostic criteria: 7.1% using DSM‐III/ICD‐10, 2.3% using DSM‐IV/DSM‐III‐R, and 5.5% in longitudinal studies using rating scales. Heterogeneity in estimates was large. Prevalence was higher among women. Lower physical activity and greater impairment were the most consistently associated risk factors. Evidence on consequences was sparse.

### 4.1 Quality of Evidence

Overall risk of bias was low, but case definition varied. Many studies reported on pure dysthymia, excluding chronic major depression and double depression, which may underestimate CD [13]. In contrast, dimensional approaches may overestimate prevalence by misclassifying depression trajectories with full remission between measurements as CD [40]. Most included studies were of high methodological quality, though older adults were sometimes underrepresented or described insufficiently. Potential subgroup analysis for groups that are also important, such as people in nursing homes or people with physical or cognitive impairments, were missing. Selection bias of healthy individuals cannot be ruled out, especially in studies with a low response rate.

### 4.2 Prevalence Among Older Adults

CD appears to be among the most common mental disorders after major depression. It may be at least prevalent as other common mental disorders in old age such as generalized anxiety disorder, posttraumatic stress disorder and alcohol use disorder [23, 75]. Assuming a chronic course in one‐third of major depression cases [8, 9, 10] and following the estimate of 7.2% for late‐life depression by Luppa et al. [2], a prevalence of 2.4% for CD would be expected. Our overall estimate of 4.0% lies higher, though the DSM‐IV‐based point prevalence of 2.3% matches this assumption.

Wang et al. [76], in a study excluded due to slightly younger sample, found that 15% of older Chinese adults remained depressed over 2 years using a CES‐D cut‐off of 10 (out of 30). In comparison, our estimate of 5.5% based on rating scales seems low, especially given meta‐analyses showing episodic late‐life depression in up to 30%–35% of older adults using rating scales [1, 3]. Most included rating scale studies utilized more conservative cut‐offs and longer timeframes than 2 years.

Impaired and institutionalized individuals were underrepresented in included studies—populations that are known to have higher rates of life‐life depression [20, 77, 78]. Therefore, the estimates reported here may underestimate the true burden of CD.

Gender‐stratified analyses showed higher prevalence in women at ratio of 1.4. This is consistent with Luppa et al. [2] reporting a ratio between 1.4 and 2.2 for episodic late‐life depression. However, categorical classifications occasionally suggested the opposite pattern, likely due to a few outlier studies [33, 49, 70].

Life‐time (3.0%) and 12‐month (2.6%) prevalence rates mostly reflect dysthymia and may underestimate total CD. The closely related estimates for both timeframes indicate that a high proportion of older adults who have ever experienced dysthymia also experienced dysthymia in the last year. Whether this is a recurrence of CD or a new onset in late life can only be answered by examining the incidence.

### 4.3 Heterogeneity

Prevalence estimates varied widely, both between classification systems and within them. This high degree of heterogeneity was expected and was also evident in other meta‐analyses on the prevalence of late‐life depression [2], even when the same instrument was used exclusively [1]. Potential reasons for this are far‐reaching and include the methodology, regional, and cultural differences, and the exact sample composition.

DSM‐III presumably included depressive symptoms regardless of severity lasting for at least 2 years as dysthymic [79], explaining substantially higher estimates. On the other hand, studies using DSM‐IV/DSM‐III‐R mostly reported dysthymia in the milder sense. Some studies using broader definitions found notably higher 12‐month and life‐time rates: [33, 43, 58].

It has also been previously discussed that standardized interviews might underestimate the prevalence of depression in older adults [34, 80] due to symptom attribution to physical illness [33, 34] and recall bias, especially for 12‐month and life‐time prevalence. Dimensional and longitudinal measures might provide complementary data.

Regional differences further contributed to heterogeneity. Generally, higher prevalence is found in higher income countries, but also in countries with economic and humanitarian crises [81]. A greater prevalence of affective disorders has been found in North America than in Europe [81, 82]. Our results of higher prevalence in Europe than in North America is therefore surprising. While this could indicate a spurious finding, Luppa et al. [2] found higher prevalence of late‐life depression in Europe as well. This could be due to cultural or biographical factors (e.g., war or post‐war experiences). Within Europe, CD prevalence varies considerably as well, with higher rates in southern and eastern countries [83]. Further research is needed.

Further, demographic differences (e.g., age‐distribution, gender‐ratio) also likely influenced estimates. Meta‐regression including this information, if available, could provide deeper insights.

### 4.4 Risk Factors and Consequences

Similar to late‐life depression [25, 84], inconsistency was found regarding age and gender. The overall prevalence pattern regarding gender aligns with findings that women experience higher rates of affective disorders across the lifespan. Possible explanations include social roles, coping styles, and women being more likely to report depressive symptoms [2]. Because women generally live longer than men, they are more likely to experience stressful life events, such as the death of someone close. The relationship between CD and age (e.g., younger old vs. oldest old) remains unclear based on our data and within the existing literature [2, 84].

Likewise, studies did not distinguish between lifelong CD and late‐onset cases, which could explain the unexpected lack of associations with marital status, living situation and socioeconomic status [20, 25]. These factors are usually linked to late‐life depression [25], but may play a smaller role in life‐long cases where genetic and early‐life factors are more influential [16].

Lower physical activity and greater impairment were consistently associated with CD, aligning with existing literature on late‐life depression [25, 84]. According to the theory of selection, optimization and compensation [85], depression may develop when individuals fail to adapt to age‐related losses—suggesting that low activity and impairment could reflect a lack of effective coping strategies.

Evidence on psychosocial factors remains limited. Psychological comorbidities (e.g., anxiety) are observed for both CD and late‐life depression [10, 20, 25]. Devanand [16] on the other hand reported frequent “pure” forms of dysthymia without psychiatric comorbidities in older adults. Substance use appears less relevant for CD in older adults. Overall, more research is needed to determine the broader clinical profile of CD in older adults.

Two included studies [33, 41] and one additional longitudinal study Comijs et al. [11] found that about half of late‐life depression cases became chronic, suggesting chronicity may be more common in older adults than in the general population. However, evidence remains too limited for firm conclusions.

Consequences of CD—such as health care use, disability and morbidity ‐ are underresearched. Nonetheless, our limited findings align with other work indicating under‐recognition and under‐treatment [16]. Associations with increased mortality [7] highlight the need for screening and treatment.

### 4.5 Strengths and Limitations

This is the first comprehensive systematic review and meta‐analysis on CD in older adults. Our extensive literature search yielded more studies than anticipated, based on previous reviews [16, 23]. We were able to conduct separate meta‐analyses for different timeframes (point, 12‐month and life‐time) and classification systems, as well as explore gender differences and sources of heterogeneity using meta‐regression.

This review also presents, for the first time, a systematic synthesis of risk factors and consequences associated with CD in late‐life. The review was pre‐registered, used a rigorous citation search, and applied independent screening and quality appraisal by two reviewers.

However, limitations include the exclusion of gray literature, and publications in other languages (i.e., other than English, German or Polish). We included studies reporting only on dysthymia to maximize available information, but this may underestimate overall CD prevalence. Our approach of pooling across classification systems for a more comprehensive picture (see 2.7.) remains debatable in the light of comparability. However, we also reported stratified estimates by classification system. Moreover, the classification system was included in meta‐regression. Data extraction was not performed by two independent raters, but was thoroughly checked by a second rater. Quality appraisal includes a subjective component and could have been expanded using other tools than the JBI standardized critical appraisal instrument. However, this tool is widely used and recommended for prevalence studies in a recent systematic review [86]. Moreover, two raters independently assessed the study quality (if necessary, a third party was involved). Potentially associated factors were only summarized descriptively in this review. Among other things, the reported statistical models and outcomes were too different for a meta‐analytic summary.

### 4.6 Recommendations for Future Research

Future studies should adopt the comprehensive DSM‐V definition of CD, use both categorical and dimensional tools, and include all segments of the older population—including institutionalized, impaired and marginalized groups. Longitudinal, populations‐based designs are needed to clarify incidence, chronicity and consequences, especially in underrepresented regions. Regional differences in prevalence require further cross‐country comparisons. Our summary of potential associated factors clearly shows that more studies are needed and that these should be summarized quantitatively if possible.

## 5 Conclusion

CD affects about 4% of older adults worldwide and may be among the most common mental disorders after major depression. Prevalence varies widely depending on diagnostic criteria and sampling. Women, those with lower physical activity and higher impairment appear at greater risk. Despite its impact, CD remains under‐recognized and undertreated. Future research should use comprehensive definitions and include all relevant subpopulations.

## Ethics Statement

This study was conducted in accordance with the Helsinki Declaration of 1964, as revised in 2024. The study was PROSPERO preregistered: https://www.crd.york.ac.uk/PROSPERO/view/CRD42025649324.

## Consent

The authors have nothing to report.

## Conflicts of Interest

The authors declare no conflicts of interest.

## Permission to Reproduce Material From Other Sources

The authors have nothing to report.

## Supporting information


Supporting Information S1

## Data Availability

The data and analysis syntax have been made available on OSF: Stratmann, M. W., König, H.‐H., & Hajek, A. (2025, May 7). Chronic depression among older adults. Retrieved from osf.io/ncrx9.
