# Supplementary material for: Prevalence and Associated Factors of Chronic Depression Among Older Adults: A Systematic Review, Meta‐Analysis, and Meta‐Regression
Source: Int J Geriatr Psychiatry. 2025 Oct 18;40(10):e70160. doi: 10.1002/gps.70160 (PMC12535281; doi:10.1002/gps.70160)
Supplement: Supplementary file 1 — Supporting Information S1 [file GPS-40-e70160-s001.docx]

**Supplementary Material to Article**

“Prevalence and associated factors of chronic depression among older adults: A systematic Review, meta-analysis, and meta-regression”

**Content**

[**Table 1.** MEDLINE (PubMed) Search 2](#_Toc195012402)

[**Table 2.** Web of Science 3](#_Toc195012403)

[**Table 3.** CINAHL (EBSCOhost) 4](#_Toc195012404)

[**Table 4.** PsycINFO (Ovid) 5](#_Toc195012405)

[**Table 5.** Quality assessment / Risk of bias. 6](#_Toc195012406)

[**Table 6.** Prevalence of chronic depression in older adults by gender and age 9](#_Toc195012407)

[**Table 7.** Associated factors reported 15](#_Toc195012408)

[**Table 8.** Summary of associated factors 29](#_Toc195012409)

[**Figure 1.** Random-effects meta-analysis for 12 month prevalence of chronic depression in older adults by diagnostic system 30](#_Toc195012410)

[**Figure 2.** Random-effects meta-analysis for life-time prevalence of chronic depression in older adults by diagnostic system 31](#_Toc195012411)

[**Figure 3.** Funnel plot for studies on point prevalence 32](#_Toc195012412)

# **Table 1.** MEDLINE (PubMed) Search

| **Search number** | **Search terms** | **Results** |
| --- | --- | --- |
| **#1** | "Chronic Depression"[All Fields] OR "Chronic depressive disorder"[All Fields] OR "Chronic major depression"[All Fields] OR ("dysthymias"[All Fields] OR "dysthymic disorder"[MeSH Terms] OR ("dysthymic"[All Fields] AND "disorder"[All Fields]) OR "dysthymic disorder"[All Fields] OR "dysthymia"[All Fields]) OR "Persistent Depressive Disorder"[All Fields] OR "double depression"[All Fields] OR "persistent major depression"[All Fields] OR "persistent depressive episode"[All Fields] OR "persistent depression"[All Fields] | 5,375 |
| **#2** | "aged"[MeSH Terms] OR "aged"[MeSH Terms] OR "aged"[All Fields] OR "older adults"[All Fields] OR "elderly"[All Fields] OR "elderlies"[All Fields] OR "el-derly s"[All Fields] OR "elderlys"[All Fields] OR "elder-ly community"[All Fields] OR “60 years or older”[All Fields] OR “60 years and older”[All Fields] | 6,382,258 |
| **#3** | "epidemiology"[MeSH Subheading] OR "epidemiolo-gy"[All Fields] OR "prevalence"[All Fields] OR "preva-lence"[MeSH Terms] OR "prevalance"[All Fields] OR "prevalences"[All Fields] OR "prevalence s"[All Fields] OR "prevalent"[All Fields] OR "prevalently"[All Fields] OR "prevalents"[All Fields] OR "prevalence rate"[All Fields] OR "prevalence rates"[All Fields] OR "epidemi-ologies"[All Fields] OR "epidemiology"[MeSH Sub-heading] OR "epidemiology"[All Fields] OR "epidemi-ology"[MeSH Terms] OR "epidemiology s"[All Fields] | 3,897,811 |
| **#4** | #1 AND #2 AND #3 | 1,208 |

# **Table 2.** Web of Science

| **Search number** | **Search terms** | **Results** |
| --- | --- | --- |
| **#1** | (ALL="Chronic Depression" OR (ALL=dysthymias OR ALL="dysthymic disorder" OR (ALL=dysthymic AND ALL=disorder) OR ALL="dysthymic disorder" OR ALL=dysthymia) OR ALL="Persistent Depressive Disorder" OR ALL="double depression") | 5,507 |
| **#2** | (ALL=aged OR ALL=aged OR ALL=aged OR ALL="older adults" OR ALL=elderly OR ALL=elderlies OR ALL="elderly s" OR ALL=elderlys) | 5,141,746 |
| **#3** | (ALL=epidemiology OR ALL=epidemiology OR ALL=prevalence OR ALL=prevalence OR ALL=prevalance OR ALL=prevalences OR ALL="prevalence s" OR ALL=prevalent OR ALL=prevalently OR ALL=prevalents OR ALL=epidemiologies OR ALL=epidemiology OR ALL=epidemiology OR ALL=epidemiology OR ALL="epidemiology s") | 2,177,243 |
| **#4** | #1 AND #2 AND #3 | 728 |

# **Table 3.** CINAHL (EBSCOhost)

| **Search number** | **Search terms** | **Results** |
| --- | --- | --- |
| **#1** | ("Chronic Depression" OR "Chronic depressive disorder" OR "Chronic major depression" OR (dysthymias OR (MH "dysthymic disorder+") OR (dysthymic AND disorder) OR "dysthymic disorder" OR dysthymia) OR "Persistent Depressive Disorder" OR "double depression" OR "persistent major depression" OR "persistent depressive episode" OR "persistent depression") |  |
| **#2** | ((MH aged+) OR (MH aged+) OR aged OR "older adults" OR elderly OR elderlies OR "elderly s" OR elderlys OR "elderly community" OR "60 years or older" OR "60 years and older") |  |
| **#3** | ((MW epidemiology) OR epidemiology OR prevalence OR (MH prevalence+) OR prevalance OR prevalences OR "prevalence s" OR prevalent OR prevalently OR prevalents OR "prevalence rate" OR "prevalence rates" OR epidemiologies OR (MW epidemiology) OR epidemiology OR (MH epidemiology+) OR "epidemiology s") |  |
| **#4** | #1 AND #2 AND #3 | 288 |

# **Table 4.** PsycINFO (Ovid)

| **Search number** | **Search terms** | **Results** |
| --- | --- | --- |
| **#1** | Persistent Depressive Disorder/ or (Dysthymic Disorder/ or Late Life Depression/) or "chronic depression".mp. or "dys-thymia".mp. | 5,641 |
| **#2** | Aging/ or Geriatric Patients/ or Geriatrics/ or Older Adult-hood/ or Age Differences/ or "aged".mp. or "elderly".mp. or "older adults".mp. | 810,593 |
| **#3** | Epidemiology/ or "prevalence".mp. or "prevalent".mp. | 226,610 |
| **#4** | #1 AND #2 AND #3 | 539 |

# **Table 5.** Quality assessment / Risk of bias.

| Author (Year) | Items | | | | | | | | | Total quality score |
| --- | --- | --- | --- | --- | --- | --- | --- | --- | --- | --- |
|  | 1 | 2 | 3 | 4 | 5 | 6 | 7 | 8 | 9 |  |
| Andreas et al. (2017) / Andreas et al. (2019) | Y | Y | Y | Y | N | Y | Y | Y | N | 7 |
| Beekman et al. (2001) | Y | Y | Y | Y | N | Y | Y | Y | Y | 8 |
| Beekman et al. (2004) | Y | Y | Y | Y | U | Y | Y | Y | Y | 8 |
| Bendayan et al. (2020) - HRS | Y | Y | Y | Y | U | Y | Y | Y | U | 7 |
| Bendayan et al. (2020) - SHARE | Y | Y | Y | Y | U | Y | Y | Y | U | 7 |
| Blanco et al. (2010) | Y | Y | Y | N | U | Y | Y | Y | Y | 7 |
| Bland et al. (1988) | Y | Y | Y | N | Y | Y | Y | Y | Y | 8 |
| Byers et al. (2010) | Y | Y | Y | Y | U | Y | Y | Y | Y | 8 |
| Carta et al. (1995) | Y | Y | N | N | U | Y | Y | N | Y | 5 |
| Chong et al. (2012) | Y | Y | N | N | Y | Y | Y | Y | Y | 7 |
| Costa et al. (2007) | Y | Y | N | Y | U | Y | Y | Y | Y | 7 |
| Fichter et al. (1995) | Y | Y | N | Y | U | Y | Y | Y | Y | 7 |
| Forsell et al. (1998) | Y | Y | Y | Y | U | Y | Y | Y | Y | 8 |
| Heok et al. (1996) | Y | Y | Y | Y | U | Y | Y | Y | Y | 8 |
| Huang et al. (2019) | Y | Y | Y | N | U | Y | Y | Y | Y | 7 |
| Ihara et al. (1998) | Y | Y | Y | Y | Y | Y | Y | Y | Y | 9 |
| Isometsä et al. (1997) | Y | Y | Y | Y | N | Y | Y | Y | Y | 8 |
| Keqing et al. (2008) | Y | Y | Y | N | U | Y | Y | N | Y | 6 |
| Kivelä et al. (1989) | Y | Y | Y | Y | U | Y | Y | Y | Y | 8 |
| Kohn et al. (2008) | Y | Y | N | Y | U | Y | Y | Y | Y | 7 |
| Kramer et al. (1985) | Y | Y | Y | Y | N | Y | Y | Y | Y | 8 |
| Livne et al. (2018) | Y | Y | Y | N | U | Y | Y | Y | N | 6 |
| Lobo et al. (1995) | Y | Y | Y | Y | Y | Y | Y | Y | Y | 9 |
| Madianos et al. (1992) | Y | Y | N | Y | Y | Y | Y | Y | Y | 8 |
| Markkula et al. (2015) | Y | Y | Y | Y | U | Y | Y | Y | Y | 8 |
| Park et al. (2015) | Y | Y | N | Y | U | Y | Y | Y | N | 6 |
| Peltzer et al. (2022) | Y | Y | Y | Y | U | Y | Y | Y | Y | 8 |
| Pengpid et al. (2023) | Y | Y | Y | Y | U | Y | Y | Y | N | 7 |
| Penninx et al. (1998) | Y | Y | Y | Y | U | Y | Y | Y | Y | 8 |
| Phillips et al. (2009) | Y | Y | Y | N | N | Y | Y | Y | Y | 7 |
| Regier et al. (1988) | Y | Y | Y | N | U | Y | Y | Y | Y | 7 |
| Reynolds et al. (2015) | Y | Y | Y | Y | U | Y | Y | Y | Y | 8 |
| Schoevers et al. (2003) | Y | Y | Y | N | U | Y | Y | Y | N | 6 |
| Skoog et al. (1993) | Y | Y | N | Y | U | Y | Y | Y | Y | 7 |
| Subramaniam et al. (2020) | Y | Y | Y | N | U | Y | Y | Y | N | 6 |
| Teng et al. (2013) | Y | Y | Y | N | U | Y | Y | Y | Y | 7 |
| Wells et al. (2006) | Y | Y | U | N | U | Y | Y | N | Y | 5 |
| Zheng et al. (2018) | Y | Y | Y | Y | U | Y | Y | Y | Y | 8 |
| **Note**. The Joanna Briggs Institute (JBI) standardized critical appraisal instrument for prevalence studies was utilized. The criteria were as follows: 1: sample frame appropriateness; 2: participant sampling appropriateness; 3: adequacy of sample size; 4: description of study subjects and setting; 5: data analysis: sufficient coverage of the identified sample; 6: valid methods used for the identification of the condition; 7: standard and reliable measurement of condition; 8: appropriateness of analytical approach; 9: adequacy of response rate, if not: appropriate management of low response rate.  **Abbreviations**. Y: Yes; N: No; U: Unknown/not reported. | | | | | | | | | | |

# **Table 6.** Prevalence of chronic depression in older adults by gender and age

| Author (Year) | Country | Sample; design | Prevalence %  (CD / n) for men | Prevalence %  (CD / n) for women | [age bracket]  prevalence %  (CD / n) |
| --- | --- | --- | --- | --- | --- |
| Andreas et al. (2017) | Europe | MentDis_ICF65+ study; cross-sectional | NR | NR | NR |
|  | Netherlands | Longitudinal Aging Study Amsterdam (LASA) |  |  |  |
| Beekman et al. (2001) |  | longitudinal | NR | NR | NR |
| Beekman et al. (2004) |  | cross-sectional | LT: 3.00  (8 / 272) | LT: 5.82 (22 / 374) | NR |
| Bendayan et al. (2020) | USA | Health and Retirement Sample (HRS); longitudinal | P: 0.9 (13 / 1397) | P: 2.9 (60 / 2069) | NR |
| Bendayan et al. (2020) | Europe | Survey of Health, Ageing and Retirement in Europe (SHARE); longitudinal | P: 3.4 (62 / 1809) | P: 10.5  (224 / 2131) | NR |
|  | European Mediterranean  (Spain, France, Italy, and Israel) |  | P: 5.0  (35 / 705) | P: 15.8 (139 / 882) | NR |
|  | non-Mediterranean  (Sweden, Denmark, Netherlands, Germany, Belgium, Switzerland, and Austria) |  | P: 2.4 (27 / 1104) | P: 6.8  (85 / 1249) | NR |
| Blanco et al. (2010) | USA | National Epidemiologic Survey on Alcohol and Related Conditions (NESARC) 2001; cross-sectional | NR | NR | NR |
| Bland et al. (1988) | Canada | NR; cross-sectional | NR | NR | NR |
| Byers et al. (2010) | USA | National Comorbidity Survey - Replication (NCS-R); cross-sectional | NR | NR | 12M:  [55-64]: 1.2 (NR / 1114)  [65-74]: 0.5 (NR / 813)  [75-84]: 0.5 (NR / 526)  [85+]: 0.5 (NR / 122) |
| Carta et al. (1995) | Italy | Health in Sardinia; cross-sectional | NR | NR | NR |
| Chong et al. (2012) | Singapore | Singapore Mental Health Study (SMHS); cross-sectional | NR | NR | NR |
| Costa et al. (2007) | Brazil | Bambuí Health Aging Study (BHAS); cross-sectional | P: 14.7  (22 / 147) | P: 6.4 (16 / 245) | P:  [75-84]: 9.0 (NR / 316) [85+]: 8.6 (NR / 76) |
| Fichter et al. (1995) | Germany | Munich Study on the oldest old; cross-sectional | 12M: 4.9 (4 / 82) | 12M: 5.1 (14 / 276) | 12M: [85-89]: 6.1 (16 / 267)  [90+]: 2.2 (2 / 91) |
| Forsell et al. (1998) | Sweden | NR; cross-sectional | P: 4.0 (10 / 247) | P: 3.4 (29 / 854) | NR |
| Heok et al. (1996) | Singapore | NR; cross-sectional | NR | NR | NR |
| Huang et al. (2019) | China | China Mental Health Survey (CHMS); cross-sectional | NR | NR | NR |
| Ihara et al. (1998) | Japan | NR; cross-sectional | P: 0.4 (3 / 809) | P: 0.5 (6 / 1156) | P: [<75]: 0.5 (6 / 1280) [>=75]: 0.4 (2 / 685) |
| Isometsä et al. (1997) | Finland | Use of Health Services and Health Status; cross-sectional | P: 1.2 (3 / 250) | P: 0.6  (2 / 333) | P: [60-79]: 0.9 (5 / 556) |
| Keqing et al. (2008) | China | NR; cross-sectional | NR | NR | P:  [60-69]: 3.59 (NR / NR)  [70+]: 3.52 (NR / NR) |
| Kivelä et al. (1989) | Finland | Ähtäri Study 1984; cross-sectional | P: 17.2 (65 / 505) | P: 22.9  (134 / 730) | P:  [60-69]: 21.6 (NR / NR) [70+]: 19.5 (NR / NR) |
| Kohn et al. (2008) | Chile | Chile Psychiatric Prevalence Study (CPSS); cross-sectional | NR | NR | LT:  [65-75]: 7.8 (18 / 226) [75+]: 3.6 (5 / 126)  12M: [65-75]: 1.9 (4 / 226) [75+]: 0.8 (1 / 126) |
| Kramer et al. (1985) | USA | Eastern Baltimore Mental Health Survey (EBMHS); cross-sectional | NR | NR | 6M: [65-74]: 1.0 (6 / 589)  [75+]: 1.1 (4 / 334) |
| Livne et al. (2018) | USA | National Epidemiologic Survey on Alcohol and Related Conditions (NESARC) 2012; cross-sectional | NR | NR | 12M:  [63-72]: 5.27 (201 / 3823) |
| Lobo et al. (1995) | Spain | Zaragoza City Study; cross-sectional | NR | NR | NR |
| Madianos et al. (1992) | Greece | NR; cross-sectional | P: 5.2  (5 / 95) | P: 5.7 (9 / 159) | P:  [65-74]: 2.7 (4 / 150)  [75+]: 10.0 (10 / 101) |
| Markkula et al. (2015) | Finland | Finnish Health 2011 Survey; cross-sectional | 12M: 6.4  (2 / NR) | 12M: 4.0  (8 / NR) | 12M:  [65-74]: 4.0 (7 / 1220)  [75+]: 6.3 (3 / 1017) |
| Park et al. (2015) | South-Korea | Yeoncheon Elderly Depression and Dementia Study (YEDD); longitudinal | P: 6.4 (9 / 141) | P: 10.6 (21 / 199) | NR |
| Peltzer et al. (2022) | South-Africa | International Network for the Demographic Evaluation of Populations and their Health (INDEPTH); longitudinal | P: 3.3  (77 / 2346) | Not included | P:  [60-69]: 3.2 (20 / 621)  [70-79]: 4.4 (21 / 475)  [80+]: 4.3 (10 / 234) |
| Pengpid et al. (2023) | Thailand | Health, Aging, and Retirement in Thailand (HART); longitudinal | P: 1.7  (27 / 1578) | P: 2.7  (49 / 1812) | P:  [55-64]: 1.9 (17 / 913)  [65-74]: 2.4 (21 / 872)  [75+]: 3.0 (29 / 976) |
| Penninx et al. (1998) | USA | Established Populations for Epidemiologic Studies of the Elderly (EPESE); longitudinal | NR | NR | NR |
| Phillips et al. (2009) | China | NR; cross-sectional | NR | NR | NR |
| Regier et al. (1988) | USA | Epidemiologic Catchment Area Survey (ECA); cross-sectional | P: 1.0 (NR / NR) | P: 2.3 (NR / NR) | NR |
| Reynolds et al. (2015) | USA | National Epidemiologic Survey on Alcohol and Related Conditions (NESARC) 2004; cross-sectional | 12M: 0.61 (40 / 4938) | 12M: 1.20  (98 / 7374) | 12M:  [55-64]: 1.34 (87 / 5135)  [65-74]: 0.67 (27 / 3634)  [75-84]: 0.54 (17 / 2673)  [85+]: 0.77 (7 / 870) |
| Schoevers et al. (2003) | Netherlands | Amsterdam Study of the Elderly (AMSTEL); longitudinal | NR | NR | NR |
| Skoog et al. (1993) | Sweden | Gothenburg City Study; cross-sectional | P: 5.6 (8 / 143) | P: 4.0 (14 / 351) | NR |
| Subramaniam et al. (2020) | Singapore | Singapore Mental Health Study 2 (SMHS-2); cross-sectional | NR | NR | LT:  [65+]: 0.2 (3 / 1297)  12M:  [65+]: 0.2 (3 / 1297) |
| Teng et al. (2013) | Taiwan | Survey of Health and Living Status of the Elderly in Taiwan; longitudinal | P: 5.1  (50 / 979) | P: 12.7  (102 / 805) | NR |
| Wells et al. (2006) | New Zealand | New Zealand Mental Health Survey (NZMHS); cross-sectional | NR | NR | 12M: [65+]: 0.4 (NR / NR) |
| Zheng et al. (2018) | England | English Longitudinal Study of Ageing (ELSA); longitudinal | NR | NR | NR |
| **Note**. Estimates of the numerators or denominators based on prevalence may result in inaccuracies. Abbreviations: LT: life-time prevalence; NR: not reported; P: Point/1-month prevalence; 6M: 6-month prevalence; 12M: 12-month prevalence. | | | | | |

# **Table 7.** Associated factors reported

| Author (year) | Study | Analysis method | Significantly associated factors | Not significantly associated factors | Other information |
| --- | --- | --- | --- | --- | --- |
| (Andreas et al., 2019) | MentDis_ICF65+ study | Logistic regressions to explore associations between 12-month dysthymia prevalence and sociodemographic factors | - gender: men (OR: 0.58 [0.36-0.94], p<.05) - age (65-69 as reference)  - >80 (OR: 0.33 [0.12-0.92], p<.05) - education in years  (OR: 0.92 [0.86-0.97], p<.01)  Comorbidities:  - any anxiety disorder  (OR: 4.17 [2.69-6.47], p<.001)  - any somatoform disorder  (OR: 6.41 [2.62-15.68], p<.001)  - posttraumatic stress disorder  (OR: 14.04 [4.70-41.8], p<.001) | - age (65-69 as reference)  - 70-74  - 75-79 - marital status  - financial situation - living situation - religious affiliation - social support - socioeconomic status   Comorbidities:  - alcohol dependence or abuse  - alcohol dependence  - any physical illness | - 58.7% of patients with dysthymia were not treated for periods of depression |
|  |  | Linear regression analysis to analyze the relation between dysthymia and measures of functional impairment, quality of life and symptom severity | - functional impairment (b=2.95 [1.00-4.91], p<.01) - quality of life (b=-13.54 [-19.45--7.63], p<.001) - symptom severity (b=0.28 [0.18-0.39], p<.001) | - disability days | - |
| (Beekman et al., 2001) | Longitudinal Aging Study Amsterdam (LASA) | Logistic regression with risk factor for persistence of depression | - chronic physical illness  (b=0.37, se=0.13, p<.004)  - external locus of control  (b=0.14, se=0.04, p<0.001) | - baseline depression level - gender: women - lower level of education - functional limitations - smaller network | - |
| (Beekman et al., 2004) | Longitudinal Aging Study Amsterdam (LASA) | Risk factors for late life dysthymia in a parsimonious multivariate logistic regression model | - smaller size network  (B=0.073, se=0.037, p=.049) - received less emotional support (B=0.061, se=0.029, p=.035) - external locus of control (B=0.31, se=0.058, p<.001) - serious events world war II (B=1.33, se=0.41, p<.001) - family history affective disorder (B=1.39, se=1.40, p<.001) | - gender: women (B=0.73, se=0.41, p=.075) | - full list of associated factors is given in the paper |
|  |  | Bivariate associations with risk factors were assessed by calculating odds ratios or by using F statistics derived from ANOVA, depending on the level of measurement of the risk factor | - lower level of education  (OR: 2.32 [1.37–3.94])  - external locus of control  (F= 77.33, df=1.381, P < 0.001)  - number of chronic illnesses  (F=13.85, df=1.381, P< 0.001)  - functional limitations  (F=13.86, df=1.380, P< 0.001)  - smaller size of the social network  (F=4.71, df=1.365, P= 0.31)  - the total number of recent adverse events (F=8.92, df=1.367, P= 0.003)  - family history of affective disorders  (OR: 4.37; [2.50–7.65])  - personal history with depression  (OR: 36.97 [17.45–78.33])  - enduring conflicts with important others  (OR: 4.22 [1.95–9.15])  - serious illness in the partner  (OR: 2.71 [1.20–6.13])  - having suffered extreme events during world war II was associated with dysthymia  (OR: 3.33 [1.77–6.25])  - having suffered serious maltreatment in early life (OR: 16.26 [3.20– 82.60])  Comorbidities: - chronic lung disease (OR: 3.73 [1.86–7.50])  - rheumatoid arthritis (OR: 2.95 [1.04–8.42]) | - marital status  (OR: 1.64 [0.97-2.77])  - living in a large city  (OR: 1.24 [0.70-2.20])  - cognitive decline  (OR: 0.59 [0.24-1.46])  - instrumental support received  (F=0.98, df=1.364, p= .32)  - emotional support received (F=0.41, df=1.364, p= .53)  - total number of catastrophic events in early life  (F=2.86, df=1.371, p= .09)  Comorbidities: - heart disease  (OR:1.15; 0.60–2.21)  - atherosclerotic disorders (OR:1.77 [0.81–3.84])  - stroke  (OR: 2.30 [0.90– 5.87])  - diabetes  (OR: 1.85 [0.82–4.18])  - osteoarthritis  (OR: 1.70 [1.00–2.90])  - cancer  (OR: 1.70 [0.76–3.80]) | - double depression: 43.6% of interviewees with dysthymia fullifled current criteria for MDD |
|  |  | Chi-Square test or Fishers test comparing characteristics of dysthymia vs. major depression | - weight loss (26.3% vs. 51.6%, p=.046) - death wishes (42.1% vs. 6.5%, p<.001) - longest depressive episode (244 weeks vs. 29 weeks, p<.001) - age of onset (31 years vs. 53 years, p<.001) | - lack of appetite - appetite increased - lack of sleep - sleep too much - lack of energy - psychomotor inhibition  - agitation - lack of libido - worthlessness - lack of concentration - slowing of thought - thinking of death - thinking of suicide - suicide attempt - number of episodes | - |
| (Bendayan et al., 2020) | Health and Retirement Sample (HRS) and Survey of Health, Ageing and Retirement in Europe (SHARE) | Chi-Square and Kruskal-Wallis tests for differences in baseline characteristics | - Persistently depressed and recovered had significantly lower baseline memory scores compared to no depression group (NR). | - | - |
| (Costa et al., 2007) | Bambuí Health Aging Study (BHAS) | Adjusted odds ratio for associations with common mental health disorders and with ‘restriction in activities of daily living’ (most restricted vs. least restricted half of the population) | - | - dysthymia (AOR: 1.72 [0.47-6.26], p=.36) | - about half of dysthymia cases were comorbid with current depressive episode - number of chronic diseases ascertained 4 years earlier was most predictive factor |
| (Fichter et al., 1995) | Munich Study on the oldest old | Univariate analyses (logistic regression) for correlates with demographic information | - | - demographic factors - cognitive impairment | - |
| (Forsell et al., 1998) | NR | Chi-Square test for differences in dysthymia prevalence | - in demented persons vs. non-demented (5.9% vs. 2.6%, p<.01) | - | - of 39 persons with dysthymia, 25 had consulted a physician during the last two weeks - 2 persons with dysthymia were treated with antidepressants |
| (Ihara et al., 1998) | NR | Chi-Square test for differences in prevalence | - | - gender (p=.964) - agegroup (p=.757) | - |
| (Kivelä et al., 1989) | Ähtäri Study 1984 | Odds ratio of dysthymic disorder of women related to men (reference) | - age: 70+ (OR: 1.8 [NR-NR], p<.05) - marital status: married (OR: 1.5 [NR-NR], p<.05) - education: basic compulsory or more (OR: 1.6 [NR-NR], p<.05) | - age: 60-69 - marital status  - unmarried  - widowed  - divorced - education: less than basic compulsory - occupation: agriculture or independent, service, industry or construction - form of social and health care: independently at home, home nursing, long-term institution | - disorder duration: difference between men and women in the mean of duration of dysthymic disorder was not significant (p=.274)  - diagnosed before study: only 13 (20%) of men and 35 (26%) of women with dysthymia were diagnosed with it earlier |
| (Kohn et al., 2008) | Chile Psychiatric Prevalence Study (CPSS) | - | - | - | - 39 cases (25.6%) with dysthymia had age of disorder onset after age 59 (N = 515) |
| (Park et al., 2015) | Yeoncheon Elderly Depression and Dementia Study (YEDD) | Adjusted odds ratios from multivariate logistic regression models for independent risk factors of persistent depression during 5 years | - physical activity (no as reference)  - above-moderate (AOR: 0.17 [0.03-0.92], p<.05) | - alcohol (AOR: 0.23 [0.03-2.1] - smoking (AOR: 10.55 [0.83-133.77]) - physical activity (no as reference)  - mild (AOR: 0.16 [0.01-2.02]) - chronic medical illnesses (0 as reference)  - 1-2 (AOR: 3.44 [0.50-23.52])  - >=3 (AOR: 12.74 [0.69-234.50]) - cognitive function  - MMSE (AOR: 0.81 [0.62-1.06])  - Initiation/Perservation subscale of the Dementia Rating Scale (AOR: 1.00 [0.88-1.13]) - depressive symptoms (AOR: 1.04 [0.69-1.56]) | - |
|  |  | Baseline characteristics between patients with remitted depression vs. patients with chronic depression. Chi-Square-Tests; Student’s t test | None | - age - gender: women - years of education - marital status: widowed/divorced/ separated/never married - living situation: living alone - Medicaid - unemployed - alcohol - smoking - physical activity - chronic medical illnesses - cognitive function - depressive symptoms | - |
| (Peltzer and Pengpid, 2022) | International Network for the Demographic Evaluation of Populations and their Health (INDEPTH) | Odds ratio for associations between chronic conditions, lifestyle factors and persistent depressive symptoms | - education in years (none as reference)  - 1-7 years (AOR: 0.45 [0.27-0.74], p<.01)  - 8-11 years (AOR: 0.20 [0.07-0.54], p<.01) - alcohol dependence (AOR: 4.54 [1.05-19.66], p<.05) | - age in years - country of birth - education in years (none as reference)  - 12 or more (AOR: 0.63 [0.26-1.54]) - marital status - wealth index - HIV positive - cardiovascular disease: yes  (AOR: 2.40 [1.02-5.35]) - hypertension - diabetes - dyslipidemia - anemia - kidney disease - current tobacco use - physical activity - fruit and vegetable intake - BMI | - |
| (Pengpid et al., 2023) | Health, Aging, and Retirement in Thailand (HART) | Odds ratio for associations between chronic conditions and persistent depressive symptoms | - social participation: yes (AOR: 0.48 [0.26-0.87], p<.05) - cardiovascular disease (AOR: 1.55 [1.01-2.39], p<.05) - number of chronic conditions: (0 as reference)  - 3 or more (AOR: 2.47 [1.07-5.67], p<.05) | - age - gender - education - marital status - religion - subjective economic status: low as reference  - middle (AOR: 0.59 [0.30-1.14])  - high (AOR: 0.46 [0.21-1.05]) - alcohol use - smoking - physical activity - BMI - diabetes - musculoskeletal disease - liver or kidney disease - respiratory disease - cancer - sensory disease - neurological disease - number of chronic conditions (0 as reference)  - 1 (AOR: 1.74 [0.86-3.52])  - 2 (AOR: 1.49 [0.68-3.29]) | - |
| (Penninx et al., 1998) | Established Populations for Epidemiologic Studies of the Elderly (EPESE) | Baseline characteristics between patients with chronic depression vs. patients with no depression Chi-Square-Tests for categorical variables; Student’s t test or Mann-Whitney test for comparison of means. All tests were two-sided. | - mean age (80.7 (5.9) vs. 78.9 (5.8), p<.001)  - gender: women (85.6% vs. 63.9%, p<.001)  - ethnicity: black (0.9% vs. 5.3%, p<.05)  - cigarette smoking: individuals with CD smoke less (p<.05)  - alcohol intake: >=28.35g/day (0.0% vs. 5.4%, p<.01)  - Physical disability (p<.001)  - no disability (13.0% vs. 52.3%)  - mobility disability (55.7% vs. 33.5%)  - mobility and disability in activities in daily life (31.3% vs. 14.2%)  - number of hospital admissions during follow-up (1.96 (2.7) vs. 1.25 (2.0), p<.001)  - use of antidepressants (15.1% vs. 4.3%, p<.001) | - BMI (24.4 (7.0) vs. 24.6 (6.3), p=.71) | - |
| (Schoevers et al., 2003) | Amsterdam Study of the Elderly (AMSTEL) | Logistical regression with persistent depressive symptoms as outcome | - personal history of depression (OR: 1.86, p=.037) - ADL disability (OR: 2.20, p=.007) - lower education (OR: 0.56, p=.030) - interaction of ‘personal history of depression’ with ‘gender’ (p=.046) | - age - gender - marital status - instrumental support - professional support - family history - chronic disease - organic syndrome - anxiety syndrome | - |
|  |  | Bivariate associations of chronicity with independent variables assessed calculating relative risks. When 95% confidence interval did not include 1, this was regarded as significant.  (no chronic depression as reference) | Vulnerability:  - personal history of depression  (OR: 1.35 [1.06-1.71])  Physical / functional stress  - ADL disability (OR: 1.44 [1.14-1.83])  Changes T0-T1; life events  Psychiatric comorbidity - new anxiety syndrome (OR: 1.64 [1.38-1.95]) | Vulnerability:  - family history of psychiatric disorder  (OR: 1.07 [0.79-1.45])  Demographic variables  - age: >74 years  (OR: 0.96 [0.75-1.23]) - gender: women  (OR: 0.92 [0.92-1.27]) - education: primary school or less  (OR: 0.84 [0.65-1.08]) - socioeconomic status: low (OR: 1.04 [0.81-1.34]) - marital status: unmarried/divorced/widowed  (OR: 0.90 [0.70-1.16]) - instrumental support: help from relatives/friends  (OR: 1.24 [0.96-1.59]) - professional support: home-care/day-care  (OR: 1.23 [0.96-1.58])  Physical / functional stress  - chronic disease (OR: 1.07 [0.83-1.39]) - IADL disability (OR: 1.28 [0.99-1.64])  Changes T0-T1; life events  - partner died (OR: 0.91 [0.57-1.46]) - relocation  (OR: 0.93 [0.68-1.29]) - new ADL disability (OR: 0.94 [0.71-1.24]) - new IADL disability  (OR: 1.06 [0.76-1.48]) - new chronic disease (OR: 1.14 [0.87-1.50]) - ADL functional improvement (OR: 1.30 [0.96-1.78]) - IADL functional improvement (OR: 0.87 [0.45-1.69])  Psychiatric comorbidity - organic syndrome (OR: 1.02 [0.67-1.56]) - low MMSE (<24) (OR: 1.04 [0.71-1.53]) - anxiety syndrome (OR: 1.20 [0.93-1.54]) - new organic syndrome (OR: 1.13 [0.85-1.51]) | Table 3 in publication gives results stratified by personal history of depression or not and by gender |
| (Skoog et al., 1993) | Gothenburg City Study | Differences in proportions were tested with Fisher’s exact test and differences in means with t-test. Associations with psychotropic drug use  (no mental disorder vs. dysthymia) | - Anxiolytics (11.5% vs. 31.8%, p<0.05)  - Anxiolytics-sedatives (26.0% vs. 50.0%, p<0.05) | - Anxiolytics-sedatives (18.1% vs. 31.8%)  - Antidepressants (2.6% vs. 9.1%)  - Neuroleptics (3.5% vs. 0%)  - Any psychotropic drug (29.1% vs. 50.0%) | - |
| (Teng et al., 2013) | Suvey of Health and Living Status of the Elderly in Taiwan | Hazard ratios for 4 year mortality  adjusted for:  - age  - gender  - education  - marital status  - smoking  - alcohol  - exercise  - BMI  - medical conditions  - self-related health - functional impairment  (no depression as reference) | - overall effect for both gender  (OR: 1.66 [1.18-2.32])  - effect for women  (OR: 1.77 [1.08-2.88])  - effect on CVD mortality for men  (OR: 2.71 [1.03-7.14])  - effect on non-CVD mortality for women  (OR: 1.96 [1.13-3.41]) | - no effect for men  (OR: 1.38 [0.84-2.26])  - no effect on non-CVD mortality for men  (OR: 1.26 [0.70-2.26])  - no effect on CVD mortality for women  (OR: 1.47 [0.48-4.53]) | - |
|  |  | Chi-square tests comparing baseline characteristics between patients with no depression vs. patients with persistent depressive symptoms | For men:  - education: illiterate (11.4% vs. 22.0%, p<.01)  - living situation: live with spouse (74.1% vs. 46.0%, p<.01)  - regular exercise (77.0% vs. 48.0%, p<.001)  - normal BMI (68.8% vs. 68.0%, p<.01)  - hypertension (40.1% vs. 56.0%, p<.05)  - diabetes (12.2% vs. 22.0%, p<.01)  - heart disease (19.9% vs. 36.0%, p<.001)  - lung disease (14.0% vs. 34.0%, p<.0001)  - cognitive impairment (2.7% vs. 16.0%, p<.0001)  - functional disability (4.2% vs. 24.0%, p<.0001)  - well self-rated health (76.5% vs. 28.0%, p<.0001)  For women:  - education: illiterate (47.5% vs. 62.8%, p<.01)  - living situation: live with spouse (43.6% vs. 29.4%, p<.05)  - regular exercise (70.5% vs. 39.2%, p<.0001)  - diabetes (16.9% vs. 31.4%, p<.01)  - heart disease (24.5% vs. 38.2%, p<.01)  - cognitive impairment (13.7% vs. 31.4%, p<.0001)  - functional disability (8.5% vs. 37.3%, p<.0001)  - well self-rated health (70.3% vs. 15.7%, p<.0001) | For men:  - mean age (76.7(4.8) vs. 77.6(5.0), p=.2467)  - current smoker (27.0% vs. 38.0%, p=.00780)  - alcohol use (36.4% vs. 22.0%, p=.0684)  - stroke (7.3% vs. 12.0%, p=.3147)  - cancer (3.2% vs. 0.0%, p=.1240)  For women:  - mean age (76.6(5.0) vs. (77.0(4.9), p=.3591)  - current smoker (3.7% vs. 4.9%, p=.5190)  - alcohol use (8.0% vs. 3.9%, p=.5540)  - normal BMI (62.0% vs. 62.8%, p=.8904)  - hypertension (48.4% vs. 50.0%, p=.9449)  - stroke (4.6% vs. 9.8%, p=.2167)  - cancer (2.8% cs. 3.9%, p=.2408)  - lung disease (9.5% vs. 17.7%, p=.1058) | - |
| (Zheng et al., 2018) | English Longitudinal Study of Ageing (ELSA) | Chi-square tests comparing baseline characteristics between participants with 0 vs. 1 vs. 2 waves of depressive symptoms | - age (65.0 (10.0) vs. 65.5 (10.4) vs. 66.6 (11.1), p<.001)  - gender: women (53.8% vs. 67.5% vs. 70.1%, p<.001)  - BMI (27.8 (4.2) vs. 28.2 (4.9) vs. 28.5 (4.7), p<.001)  - education: A-level (35.9% vs. 25.5% vs. 18.5%, p<.001)  - living situation: living alone (28.4% vs. 43.4% vs. 58.7%, p<.001)  - current smoking (13.4% vs. 18.4% vs. 27.4%, p<.001)  - alcohol drinking (59.2% vs. 44.8% vs. 36.2%, p<.001)  - antidepressant medication (0.8% vs. 3.4% vs. 5.1%, p<.001)  - diabetes (6.9% vs. 9.6% vs. 11.2%, p<.001)  - coronary heart disease (5.9% vs. 8.5% vs. 14.5%, p<.001)  - stroke (2.1% vs. 2.9% vs. 6.9%, p<.001)  - chronic lung disease (4.0% vs. 6.5% vs. 12.0%, p<.001)  - global cognitive score (35.0 (8.5) vs. 33.0 (8.8) vs. 30.0 (8.7), p<.001)  - memory score (10.5 (3.4) vs. 9.7 (3.6) vs. 8.8 (3.7), p<.001)  - executive function scores (20.7 (6.4) vs. 19.5 (6.3) vs. 17.5 (6.1), p<.001)  - orientation score (3.79 (0.48) vs. 3.76 (0.54) vs. 3.68 (0.61), p<.001)  - CES-D scores at wave 1 (0.74 (0.93) vs. 3.26 (2.18) vs. 5.61 (1.39), p<.001)  - CES-D scores at wave 2 (0.78 (0.93) vs. 3.42 (2.11) vs. 5.75 (1.36), p<.001) | - systolic blood pressure (136.2 (17.5) vs. 135.2 (17.2) vs. 136.6 (17.1), p=.589)  - diastolic blood pressure (75.7 (10.1) vs. 75.1 (10.5) vs. 75.4 (10.3), p=.084)  - hypertension (41.6% vs. 41.9% vs. 43.6%, p=.391)  - cancer (5.1% vs. 5.6% vs. 5.3%, p=.588) | - |
| Abbreviations: ADL: Activities of Daily Living scale; AOR: adjusted odds ratio; b: beta; BMI: Body Mass Index; IADL: Instrumental Activities of Daily Living scale; MMSE: Mini Mental State Examination; NR: not reported; se: standard error; OR: odds ratio. | | | | | |

# **Table 8.** Summary of associated factors

| Category | Factor | Associated* | Not Associated | Summary |
| --- | --- | --- | --- | --- |
| Socio-demographic |  |  |  |  |
|  | Age | Older age: (Andreas et al., 2019)  Younger age: (Penninx et al., 1998, Zheng et al., 2018) | (Ihara et al., 1998, Park et al., 2015, Peltzer and Pengpid, 2022, Pengpid et al., 2023, Schoevers et al., 2003, Teng et al., 2013) | Inconclusive |
|  | Gender | Women: (Andreas et al., 2019, Penninx et al., 1998) | (Beekman et al., 2001, Beekman et al., 2004, Ihara et al., 1998, Pengpid et al., 2023, Schoevers et al., 2003) | Inconclusive |
|  | Education | Lower education level: (Andreas et al., 2019, Beekman et al., 2004, Peltzer and Pengpid, 2022, Teng et al., 2013, Zheng et al., 2018)  Higher education level: (Kivelä et al., 1989, Schoevers et al., 2003) | (Beekman et al., 2001, Park et al., 2015, Pengpid et al., 2023) | Inconclusive |
|  | Socioeconomic status |  | (Andreas et al., 2019, Pengpid et al., 2023, Schoevers et al., 2003) | No |
|  | Marital status |  | (Andreas et al., 2019, Beekman et al., 2004, Park et al., 2015, Peltzer and Pengpid, 2022, Pengpid et al., 2023, Schoevers et al., 2003) | No |
|  | Living situation | Living alone: (Teng et al., 2013, Zheng et al., 2018) | (Andreas et al., 2019, Park et al., 2015) | Inconclusive |
|  | Religious affiliation |  | (Andreas et al., 2019, Pengpid et al., 2023) | Rather no |
|  | Ethnicity | White American: (Penninx et al., 1998) |  | Inconclusive |
| Health behaviour |  |  |  |  |
|  | Physical exercise | Less active: (Park et al., 2015, Teng et al., 2013) |  | Rather yes |
|  | BMI | Abnormal BMI (for men): (Teng et al., 2013) | (Pengpid et al., 2023)  Abnormal BMI (for women): (Teng et al., 2013) | Inconclusive |
|  | Alcohol | Drinkers:  (Penninx et al., 1998, Zheng et al., 2018) | (Teng et al., 2013, Park et al., 2015, Pengpid et al., 2023) | Inconclusive |
|  | Smoking | No Smoking: (Penninx et al., 1998)  Smoking: (Zheng et al., 2018) | (Teng et al., 2013, Park et al., 2015, Pengpid et al., 2023) | Inconclusive |
| Psychosocial factors |  |  |  |  |
|  | Social support | Less social or emotional support: (Beekman et al., 2004, Pengpid et al., 2023) | (Andreas et al., 2019) | Inconclusive |
|  | family history of depression | Presence of depression in family history: (Beekman et al., 2004) | (Schoevers et al., 2003) | Inconclusive |
|  | Personal history of depression | Presence of depression in personal history: (Schoevers et al., 2003) |  | Inconclusive |
|  | Traumatic experiences | Presence of traumatic experiences: (Beekman et al., 2004) |  | Inconclusive |
|  | External locus of control | (Beekman et al., 2001) |  | Inconclusive |
| Health related factors |  |  |  |  |
|  | Impairment severity | Higher impairment:  (Andreas et al., 2019, Penninx et al., 1998, Schoevers et al., 2003, Teng et al., 2013) |  | Yes |
|  | Self-rated health | Poorer self-rated health: (Teng et al., 2013) |  | Inconclusive |
|  | Quality of life | Poorer quality of life: (Andreas et al., 2019) |  | Inconclusive |
| Comorbidities |  |  |  |  |
|  | Depression severity | Higher depression score: (Andreas et al., 2019, Zheng et al., 2018) | (Beekman et al., 2001) | Inconclusive |
|  | Double Depression | High comorbidity with major depressive disorder: (Beekman et al., 2004, Costa et al., 2007) |  | Rather yes |
|  | Anxiety disorder | (Andreas et al., 2019) New anxiety disorder: (Schoevers et al., 2003) | Anxiety syndrome: (Schoevers et al., 2003) | Rather yes |
|  | Somatoform disorder | (Andreas et al., 2019) |  | Inconclusive |
|  | Posttraumatic stress disorder / traumatic experiences | Presence of traumatic experiences/disorder: (Andreas et al., 2019, Beekman et al., 2004) |  | Rather yes |
|  | Dementia / cognitive decline | Higher impairment:  (Bendayan et al., 2020, Forsell et al., 1998, Teng et al., 2013, Zheng et al., 2018) | (Beekman et al., 2004, Fichter et al., 1995, Park et al., 2015) | Inconclusive |
|  | Alcohol use disorder |  | (Andreas et al., 2019) | Inconclusive |
|  | Diabetes | Presence of diabetes: (Teng et al., 2013, Zheng et al., 2018) | (Beekman et al., 2004, Peltzer and Pengpid, 2022, Pengpid et al., 2023) | Inconclusive |
|  | Cancer |  | (Teng et al., 2013, Zheng et al., 2018, Beekman et al., 2004) | No |
|  | Hypertension | Presence of hypertension (for men): (Teng et al., 2013) | (Peltzer and Pengpid, 2022, Zheng et al., 2018)  Presence of hypertension (for women): (Teng et al., 2013) | Inconclusive |
|  | Cardiovascular disease | (Teng et al., 2013, Zheng et al., 2018, Pengpid et al., 2023) | (Beekman et al., 2004, Peltzer and Pengpid, 2022, Teng et al., 2013) | Inconclusive |
|  | Chronic physical illness | Presence of at least one chronic disease: (Beekman et al., 2001, Zheng et al., 2018)  Higher number of chronic diseases: (Beekman et al., 2004, Costa et al., 2007, Pengpid et al., 2023) | (Schoevers et al., 2003, Park et al., 2015) | Rather yes |
| Health care use |  | Higher health care use: (Forsell et al., 1998) Poor detection and treatment of chronic depression: (Andreas et al., 2019, Kivelä et al., 1989) |  | Yes |
| Medication use |  |  |  |  |
|  | Antidepressants | Higher use: (Penninx et al., 1998, Zheng et al., 2018) | (Skoog et al., 1993)  Infrequent use: (Forsell et al., 1998) | Inconclusive |
|  | Anxiolytics | Higher use: (Skoog et al., 1993) |  | Inconclusive |
| Mortality |  | Higher risk: (Teng et al., 2013) |  | Inconclusive |
| Note. *Presence of factor or higher degree of factor is associated with higher prevalence or risk of chronic depression. | | | | |


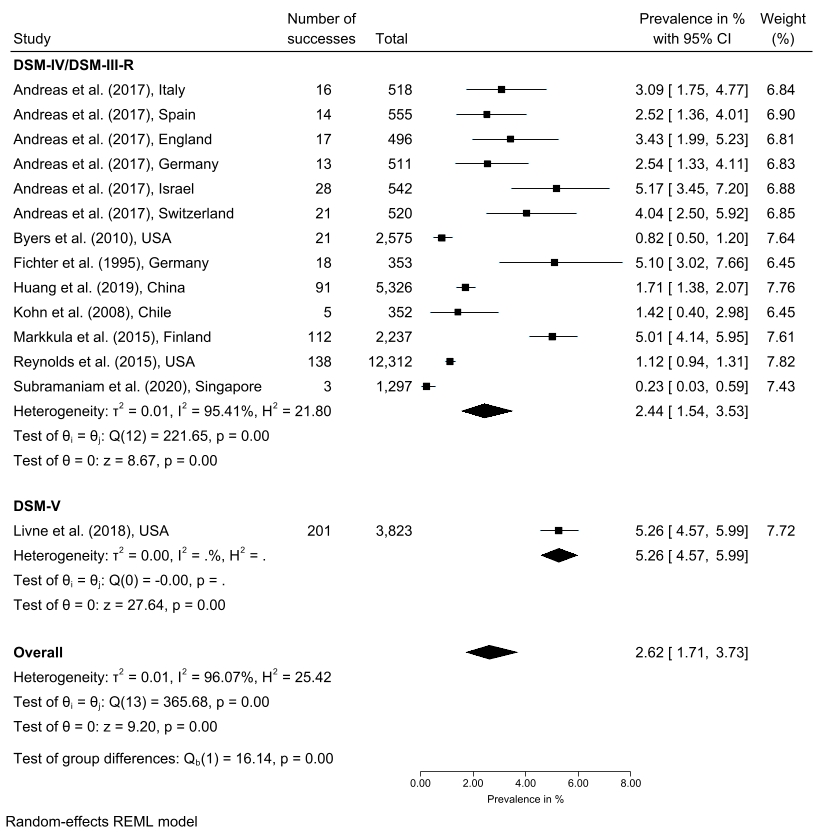


# **Figure 1.** Random-effects meta-analysis for 12 month prevalence of chronic depression in older adults by diagnostic system


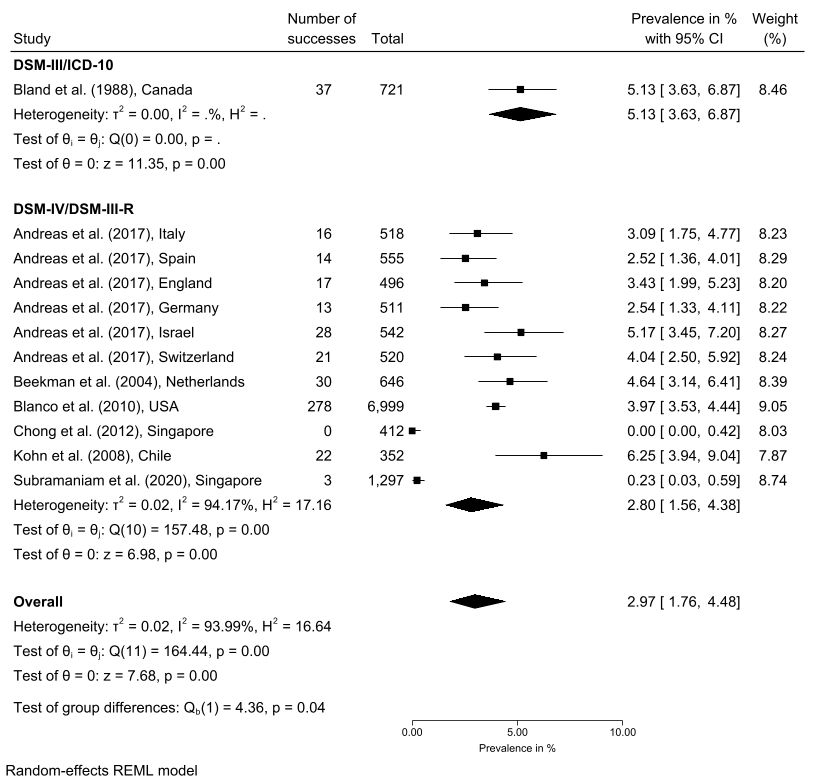


# **Figure 2.** Random-effects meta-analysis for life-time prevalence of chronic depression in older adults by diagnostic system


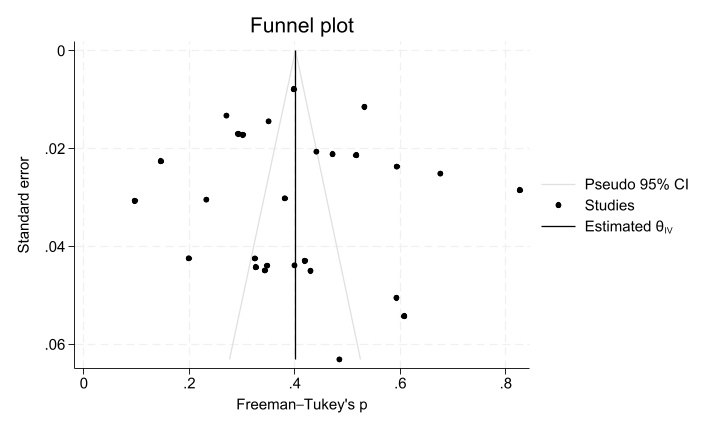


# **Figure 3.** Funnel plot for studies on point prevalence

# **References**

ANDREAS, S., DEHOUST, M., VOLKERT, J., SCHULZ, H., SEHNER, S., SULING, A., WEGSCHEIDER, K., AUSÍN, B., CANUTO, A., CRAWFORD, M. J., RONCH, C. D., GRASSI, L., HERSHKOVITZ, Y., MUÑOZ, M., QUIRK, A., ROTENSTEIN, O., SANTOS-OLMO, A. B., SHALEV, A. Y., WEBER, K., WITTCHEN, H. U. & HÄRTER, M. 2019. Affective disorders in the elderly in different European countries: Results from the MentDis_ICF65+study. *Plos One,* 14**,** 13.

BEEKMAN, A., DEEG, D., GEERLINGS, S., SCHOEVERS, R., SMIT, J. & VAN TILBURG, W. 2001. Emergence and persistence of late life depression: A 3-year follow-up of the Longitudinal Aging Study Amsterdam.

BEEKMAN, A. T. F., DEEG, D. J. H., SMIT, J. H., COMIJS, H. C., BRAAM, A. W., DE BEURS, E. & VAN TILBURG, W. 2004. Dysthymia in later life: a study in the community. *Journal of Affective Disorders,* 81**,** 191-199.

BENDAYAN, R., KELLY, A., HOFER, S. M., PICCININ, A. M. & MUNIZ-TERRERA, G. 2020. Memory Decline and Depression Onset in U.S. and European Older Adults. *J Aging Health,* 32**,** 189-198.

COSTA, E., BARRETO, S. M., UCHOA, E., FIRMO, J. O. A., LIMA-COSTA, M. F. & PRINCE, M. 2007. Prevalence of <i>International Classification of Diseases, 10th Revision</i> common mental disorders in the elderly in a Brazilian community:: The Bambui Health Ageing Study. *American Journal of Geriatric Psychiatry,* 15**,** 17-27.

FICHTER, M. M., BRUCE, M. L., SCHROPPEL, H., MELLER, I. & MERIKANGAS, K. 1995. COGNITIVE IMPAIRMENT AND DEPRESSION IN THE OLDEST-OLD IN A GERMAN AND IN US COMMUNITIES. *European Archives of Psychiatry and Clinical Neuroscience,* 245**,** 319-325.

FORSELL, Y., JORM, A. F. & WINBLAD, B. 1998. The outcome of depression and dysthymia in a very elderly population: results from a three-year follow-up study. *Aging &amp; Mental Health,* 2**,** 100-104.

IHARA, K., MURAOKA, Y., OIJI, A. & NADAOKA, T. 1998. Prevalence of mood disorders according to dsm-iii-r criteria in the community elderly residents in Japan. *Environ Health Prev Med,* 3**,** 44-9.

KIVELÄ, S.-L., PAHKALA, K., KIVELÄ, S. L. & PAHKALA, K. 1989. Dysthymic disorder in the aged in the community. *Social Psychiatry &amp; Psychiatric Epidemiology,* 24**,** 77-83.

KOHN, R., VICENTE, B., SALDIVIA, S., RIOSECO, P. & TORRES, S. 2008. Psychiatric epidemiology of the elderly population in Chile. *Am J Geriatr Psychiatry,* 16**,** 1020-8.

PARK, J. E., LEE, J. Y., KIM, B. S., KIM, K. W., CHAE, S. H. & CHO, M. J. 2015. Above-moderate physical activity reduces both incident and persistent late-life depression in rural Koreans. *Int J Geriatr Psychiatry,* 30**,** 766-75.

PELTZER, K. & PENGPID, S. 2022. Impact of Somatic Conditions and Lifestyle Behaviours on Depressive Symptoms and Low Life Satisfaction among Middle-Aged and Older Adult Men in South Africa. *Journal of Mens Health,* 18**,** 11.

PENGPID, S., PELTZER, K. & ANANTANASUWONG, D. 2023. Prevalence and determinants of incident and persistent depressive symptoms among middle-aged and older adults in Thailand: prospective cohort study. *BJPsych Open,* 9**,** e99.

PENNINX, B. W., GURALNIK, J. M., PAHOR, M., FERRUCCI, L., CERHAN, J. R., WALLACE, R. B. & HAVLIK, R. J. 1998. Chronically depressed mood and cancer risk in older persons. *J Natl Cancer Inst,* 90**,** 1888-93.

SCHOEVERS, R. A., BEEKMAN, A. T. F., DEEG, D. J. H., HOOIJER, C., JONKER, C. & VAN TILBURG, W. 2003. The natural history of late-life depression: results from the Amsterdam Study of the Elderly (AMSTEL). *Journal of Affective Disorders,* 76**,** 5-14.

SKOOG, I., NILSSON, L., LANDAHI, S. & STEEN, B. 1993. Mental Disorders and the Use of Psychotropic Drugs in an 85-Year-Old Urban Population. *International Psychogeriatrics,* 5**,** 33-48.

TENG, P. R., YEH, C. J., LEE, M. C., LIN, H. S. & LAI, T. J. 2013. Change in depressive status and mortality in elderly persons: results of a national longitudinal study. *Arch Gerontol Geriatr,* 56**,** 244-9.

ZHENG, F., ZHONG, B., SONG, X. & XIE, W. 2018. Persistent depressive symptoms and cognitive decline in older adults. *The British Journal of Psychiatry,* 213**,** 638-644.
